# A genome-wide integrated analysis of lncRNA-mRNA in melanocytes from white and brown skin hair boer goats (*Capra aegagrus hircus*)

**DOI:** 10.3389/fvets.2022.1009174

**Published:** 2022-11-03

**Authors:** Ji Kai-yuan, Zhao Yi-Wei, Wen Ru-jun, Ibrar Muhammad Khan, Zhang Yun-hai

**Affiliations:** ^1^Anhui Key Laboratory of Genetic Resources Protection and Biological Breeding for Livestock and Poultry, College of Animal Science and Technology, Anhui Agricultural University, Hefei, China; ^2^Anhui Province Key Laboratory of Veterinary Pathobiology and Disease Control, College of Animal Science and Technology, Anhui Agricultural University, Hefei, China; ^3^Anhui Province Key Laboratory of Embryo Development and Reproduction Regulation, Anhui Province Key Laboratory of Environmental Hormone and Reproduction, School of Biological and Food Engineering, Fuyang Normal University, Fuyang, China; ^4^Linquan Comprehensive Experimental Station of Anhui Agricultural University, Anhui Agricultural University, Linquan, China

**Keywords:** lncRNA, melanocyte, *Capra hircus*, genome-wide analysis, mRNA

## Abstract

Long noncoding RNAs (lncRNAs) are involved in many biological processes and have been extensively researched. Nonetheless, literature focusing on the roles of lncRNA in melanocytes is limited. Melanocytes are located in the basal layer of the epidermis and determine the color of an animal's skin and hair by producing melanin. The mechanisms of melanogenesis remain unclear. Here, melanocytes from Boer goat skins were successfully isolated and verified using morphological observation, dopamine staining, silver ammonia staining, and immunohistochemical staining *in vitro*. Phenotypic testing revealed that melanocytes isolated from goat skins with white and brown hairs showed significant differences in proliferation, migration, and melanogenesis (^**^P < 0.01). RNA sequencing was performed with the isolated melanocytes, and through bioinformatic analysis, several candidate lncRNAs and mRNAs involved in stage-specific melanogenesis were identified. Functional enrichment analysis indicated that miRNA precursors and *cis*-regulatory effects of lncRNAs were deeply dissected using the function prediction software. Multiple lncRNA–mRNA networks were presumed to be involved in melanocyte migration, proliferation, and melanogenesis based on the Kyoto Encyclopedia of Genes and Genomes (KEGG) annotation. This research provided novel bioinformatic insights into the roles of lncRNAs in mammalian pigmentation.

## Introduction

Melanoblasts are found in the skin's basal layer and migrate to the epidermis to mature into melanocytes. Mature melanocytes play roles in the skin's innate immunity and determine skin color by producing melanin (1, 2). Melanosomes are unique organelles found in melanocytes that synthesize melanin granules under tyrosinase catalysis (1). Depending on the expressed regulatory genes, melanosomes can synthesize two types of melanin granules (eumelanin and brown melanin), and the ratio of the two melanin types determines animal skin and hair color (3). Mature melanin granules can protect skin keratinocytes from UV radiation damage (4). Melanosomes contain filamentous proteins and immunoactive substances, which are considered to be special lysosomes with antigen processing and presentation capabilities (2, 5). Thus, melanocytes not only are considered adenosine cells but also participate in the skin's immune system. Although many studies have been conducted on the mechanisms underlying melanogenesis, various aspects of the process remain unclear.

More than 100 genes have been identified to regulate melanin synthesis either directly or indirectly *via* the protein kinase C (6), Wnt (7, 8), and cAMP signaling pathways (9). α-Melanocyte-stimulating hormone (α-MSH) is an endogenous neuropeptide that binds with melanocortin 1 receptor (MC1R) on melanocytes to increase cyclic adenylate (cAMP) levels (10, 11). High levels of cAMP activate tyrosinase, which catalyzes tyrosine uptake from the blood by melanocytes and melanin granule synthesis *via* melanosomes (8, 12, 13). However, mechanisms underlying melanin synthesis and its roles in immunity remain unclear.

Long noncoding RNAs (lncRNA) are transcribed by RNA polymerase II and are multiexonic, polyadenylated, >200 nt in length, and located in the nucleus or the cytoplasm (14). They can be divided into antisense (antisense long noncoding RNAs), intronic transcript (intron noncoding RNAs), intergenic (lincRNAs), and promoter-associated or UTR-associated lncRNAs (15). lncRNAs in the cytoplasm can influence protein expression (16) and act as *cis*- and *trans*-acting elements to regulate gene expression (17, 18). Recent studies implicated lncRNAs in melanogenesis (19). The ceRNA network of lncRNA and circRNA has complex interactions involving ncRNA and mRNA related to skin and melanocyte development in mice. However, the role of these lncRNAs is poorly understood.

Although it has been confirmed that lncRNAs are widely involved in disease occurrence, cell metabolism, growth, development, and other physiological processes, their role in skin pigmentation in livestock animals has not been reported. In the present study, RNA transcription sequencing technology was used to explore the expression characteristics of lncRNAs and mRNAs in melanocytes isolated from goat skins with white and brown hairs, and the lncRNA–mRNA networks were constructed using functional prediction.

## Materials and methods

### Ethics approval

The study was conducted according to the guidelines of the Declaration of Helsinki and was approved by the Ethics Committee of Anhui Agricultural University. The Institutional Animal Care and Use Committee of Anhui Agricultural University approved all animal surgeries (Approval no. 2016017).

### Primary cell collection and isolation

The experimental animals were obtained from Linquan Comprehensive Experimental Station of Anhui Agricultural University. We used three female Boer goats that were 3 months old, and adequate drinking water and feed were provided throughout the experiment to keep them in healthy condition. Skin tissues (0.8 × 0.8 cm) with white and brown hair were isolated from the backs of the goats. The skin tissues were immersed in DMEM basal medium containing penicillin (400 U/ml) and streptomycin (400 μg/ml) and cut into thin strips (0.2 × 0.8 cm) before being placed in 0.25% Dispase II for over-digestion for 12–16 h after PBS cleaning. The epidermis and dermis were separated using ophthalmic tweezers, and the separated epidermis was cut into pieces and incubated in 0.25% trypsin+0.02% EDTA at 37°C for 10 min. An organic membrane filter (Solarbio, Beijing, China) was used to filter the digested products and collect the suspended cells. The cells were resuspended in 90% DMEM+10% FBS after centrifugation (1,000 rpm/min) and incubated at 37°C in a 5% CO_2_ constant temperature incubator. After adherent growth, the cells were cultured in melanocyte medium (MelM, ScienCell Research Laboratories, Carlsbad, CA, USA) and then sub-cultured to the third generation for identification.

### Identification of melanocytes

The morphological characteristics of melanocytes were microscopically observed (Leica, Buffalo Grove, USA). Dopamine staining (1 ml KH_2_PO_4_ + 3 ml Na_2_HPO_4_ + 12.5 ml 0.2% DoPa) and silver ammonia staining (AgNO_3_ + NH_3_·H_2_O) were used to detect the presence of melanin particles in the cytoplasm of melanocytes. Briefly, melanocytes were fixed in paraformaldehyde (4%) and washed with 1 × PBS (PH 7.4). The cells were immersed in dopamine and silver ammonia stains and then incubated at 37°C for 1 h. Acidic calcium-binding protein (S100) was isolated and identified from bovine brains and is widely distributed in the cytoplasm of melanocytes, which are derived from neural crest cells (20). Immunohistochemical staining was used to detect the expression of S100 and other melanocyte markers in the cytoplasm. Melanocytes were fixed in paraformaldehyde (4%), incubated at 37°C in hydrogen peroxide (3%) for 15 min to block the action of endogenous peroxidases, and washed with 1 × PBS. The cells were immersed in bovine serum albumin (Rebiosci, Keilor, Australia) at 37°C for 25 min and incubated at 4°C overnight with rabbit primary antibodies against S100 (1:600, BioVision, San Francisco, USA). After the overnight incubation, the cells were washed with PBS (PH 7.4) and incubated with horseradish peroxidase-conjugated anti-rabbit IgG for 40 min at 37°C.

### Cell proliferation assay

In this study, a CCK8 kit (Genomeditech, Shanghai, China) was used to detect the melanocyte proliferation rate. In triplicate, 3,000 melanocytes were seeded into 96-well plates with 100 μl of complete culture medium. The melanocytes were cultured for 0, 12, 24, 36, and 48 h before 10 μl of CCK8 buffer was added to 100 μl of medium and cultured again for 3 h. The culture plates were shaken for 10 min, and the optical density (OD) values were read at 450 nm.

### Tyrosinase activity determination

Cells isolated from skins with white and brown hairs were added to 90 μl of 1% Triton X-100 and shaken for 5 min. Thereafter, 5 μl of 0.2% L-dopa was added, and the cells were incubated at 37°C for 30 min. The OD was measured at 490 nm using a microplate analyzer (Biotek, Vermont, USA), and tyrosinase activity (%) was calculated based on the OD of the treated and blank groups.

### Melanin content measurement

Melanin content was analyzed using total alkali-soluble melanin (ASM) assay. Melanocytes were collected 72 h after transfection, rinsed three times with PBS (PH 7.4), and lysed in NaOH (1 mol/L) at 37°C for 45 min. Melanin content was measured using a Multiskan Spectrum microplate reader (Biotek, Vermont, USA) using an absorbance of 475 nm. The relative melanin content was normalized based on cell number.

### Wound-healing assay

Melanocytes were seeded in 24-well cell culture plates coated with fibronectin (25 μg/well) until they grew to near confluence. A scratch was made using a pipette tip. Melanocytes were washed with PBS and cultured in an FBS-free medium. Cell migration was visualized under a microscope in a specific location at 0 h and 48 h after scratching.

### RNA isolation and LncRNA-seq

RNA samples from melanocytes were extracted using TRIzol reagent. For sequence preparation, 5 μg of RNA from each sample was used as the input, and ribosomal RNA in the samples was removed using the rRNA Removal Kit (Aksomics, Beijing, China). cDNA libraries were generated using an RNA Library Kit (NEB, Beijing, China), and cDNA qualities were assessed using an Agilent Bioanalyzer 2100 system. Illumina Novaseq™ 6000 was used for sequencing after sample clustering. Using the specified GCF/001 genome/*Capra hircus* (goat)/2016 (ftp://ftp.ncbi.nlm.nih.gov/genomes/all/GCF/001/704/415/GCF_001704415.2) as a reference (21), sequence alignment and subsequent analyses were performed. Pearson correlation coefficients (22) were calculated to reflect the degree of linear correlation between two groups of samples. The StringTie transcript assembly software (23) was used to assemble the reads. The known mRNA and transcripts < 200 bp were removed, and lncRNA prediction was performed for the remaining transcripts. The coding potential calculator (CPC) (24) and the Coding-Non-Coding Index (CNCI) (25) were used as prediction software.

### Expression pattern analysis of mRNA and lncRNA

The position of the lncRNA in the genome was determined, and classified statistics for lncRNA were generated according to the comparative analysis between lncRNA sequences and the reference genome. Differential expression of mRNAs and lncRNAs between melanocytes from skins with white and brown hairs was analyzed using false discovery rate (FDR) and fold change (26). An FPKM was developed to measure the expression of genes and lncRNAs to overcome the isoform length-dependence of the read counts (27).

### Functional analysis of differentially expressed mRNAs (DEGs) and lncRNAs (DELs)

As post-transcriptional products, lncRNAs can form miRNAs *via* shearing (28). Therefore, homology analysis was used to compare whether the DELs have miRNA precursor structures. lncRNAs play crucial roles as enhancers (29) and promoters (30) to regulate adjacent coding genes. lncRNAs can regulate the expression of genes that overlap with the lncRNA transcription start or end site at distances < 100 kb (31). In the present study, we analyzed the location of DELs and mRNAs and predicted their relationship. The Gene Ontology (GO) and KEGG databases were used to analyze the functions of the DEGs and the target genes of DELs.

### Statistical analysis

The differences in cell proliferation, tyrosinase activity, cell migration, and melanin amount were determined using Fisher's protected LSD test and ANOVA. The research data were analyzed using SPSS 11.5 (Chicago, IL, USA). The results were expressed as mean ± standard deviation (SD), with a *P*-value < 0.05 indicating statistical significance.

## Results

### Identification of melanocytes

**Figure 1:**
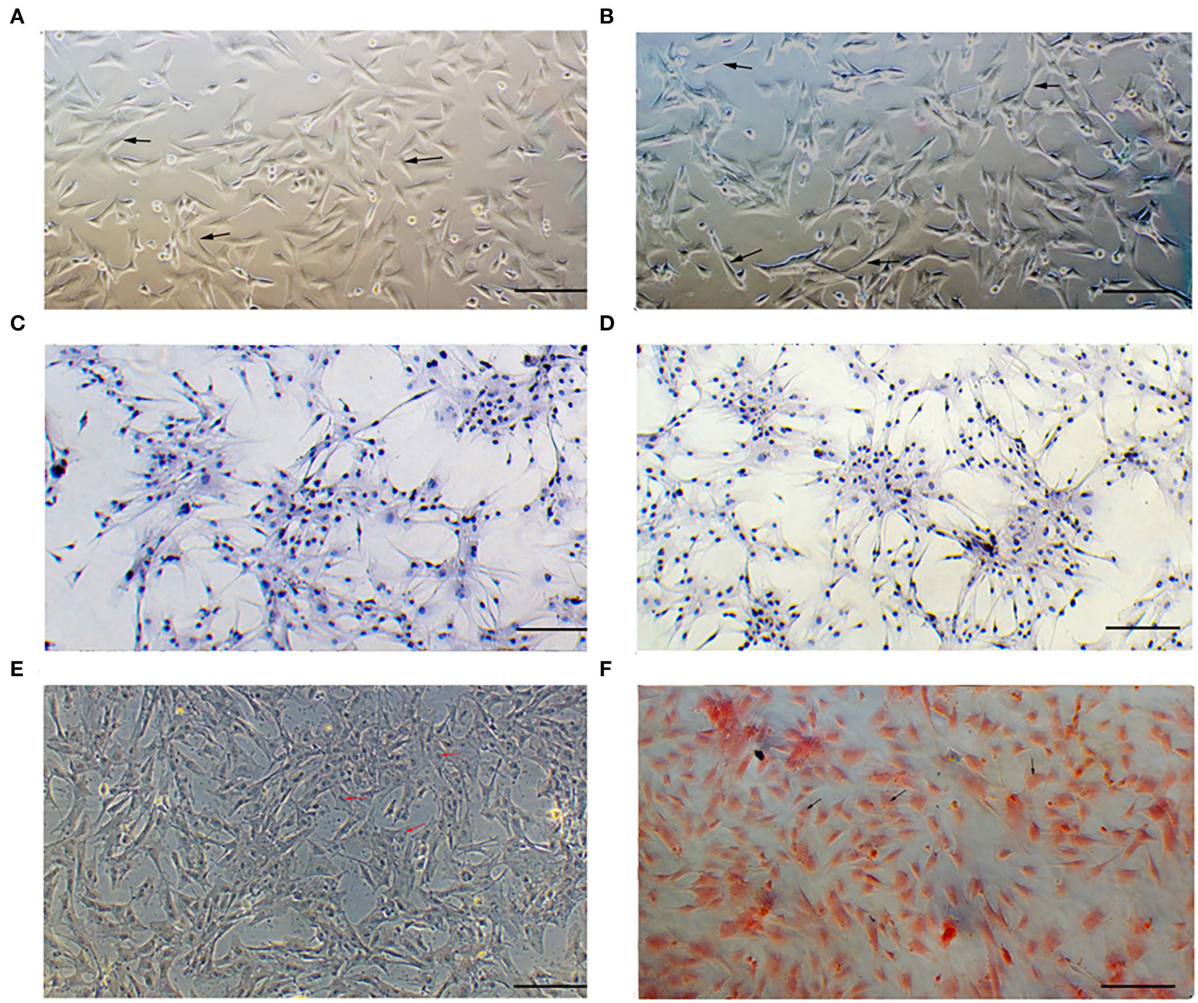
Identification of melanocytes cultured *in vitro*. **(A)** Morphology of cells isolated from skins with white hair as observed under a microscope. **(B)** Morphology of cells isolated from skins with brown hair as observed under a microscope. **(C,D)** S100 protein signaling detected by cellular immunohistochemical staining. **(E)** Melanin granules observed using dopamine staining. **(F)** Melanin granules observed using silver ammonia staining. Bar = 0.1 mm.

In the microscopic field, the cells isolated from Boer goat skins with white (Figure 1A) or brown (Figure 1B) hairs were found to be fusiform-shaped, similar to the general morphological structure of melanocytes. Immunohistochemical staining showed that S100 is detected in the cytoplasm of the cells (Figures 1C,D). Dopamine (Figure 1E) and silver ammonia staining (Figure 1F) showed melanin granules in the cytoplasm of cells.

### Melanocyte phenotypes

**Figure 2:**
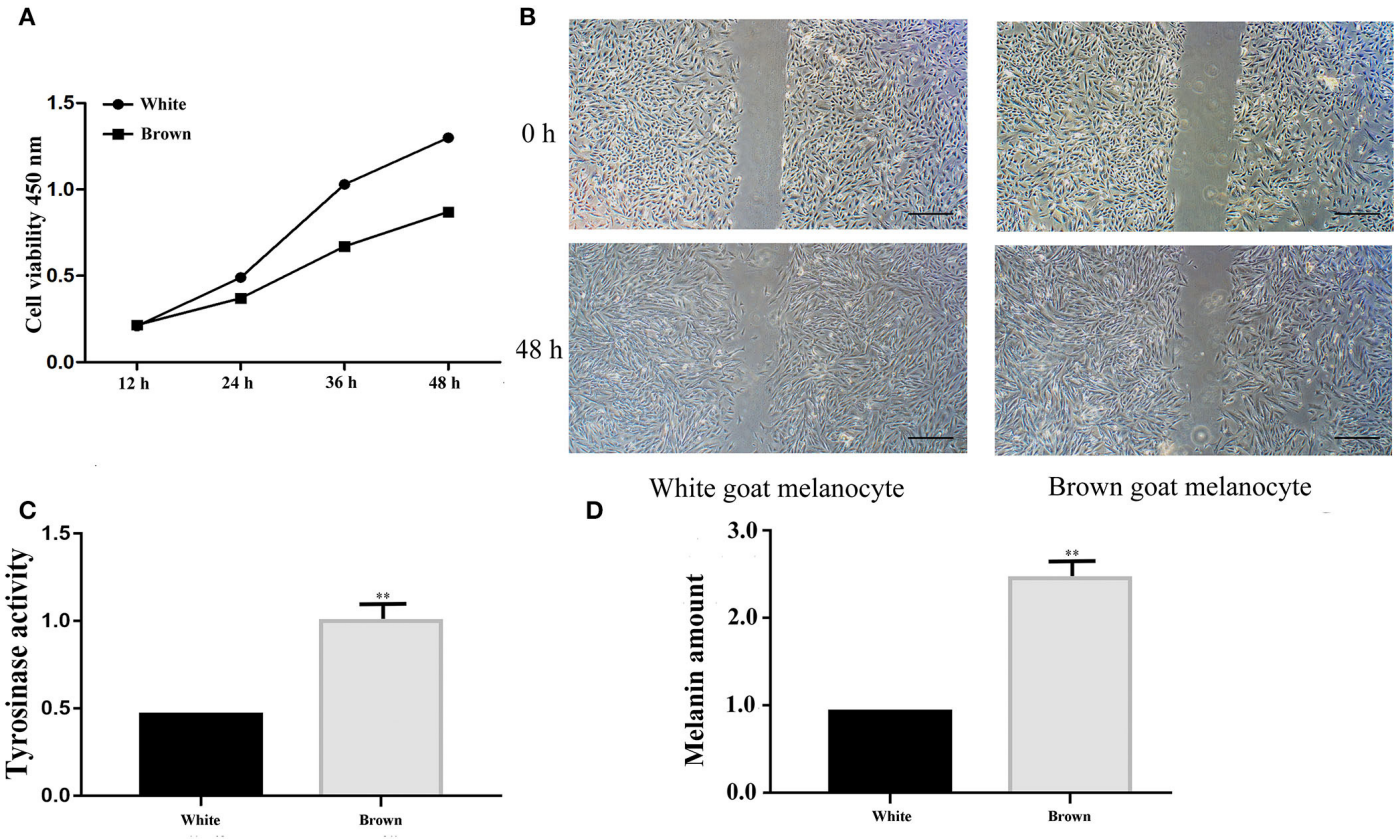
Identification of melanocyte phenotypes isolated from Boer goat skins with white and brown hairs. **(A)** Proliferation rates of melanocytes as detected by a CCK8 assay. **(B)** Migration rates of melanocytes as detected by the wound-healing assay. **(C)** Tyrosinase activity of melanocytes as detected by L-dopa staining. **(D)** Melanin production of melanocytes as detected by an ASM assay. Data are shown as the means ± SD of relative fold-change (*n* = 3 per group). **P < 0.01. Bar = 0.25 mm.

The cell proliferation assay showed that the proliferation rates of melanocytes isolated from Boer goat skins with white hair were higher than those with brown hair (Figure 2A). The migration rates of melanocytes in skins with white hair were also higher (Figure 2B). Tyrosinase activity (^**^P < 0.01; Figure 2C), as well as melanin production rates (^**^P < 0.01; Figure 2D), was significantly higher in melanocytes isolated from skins with brown hair.

### Expression patterns of lncRNAs and mRNAs

**Figure 3:**
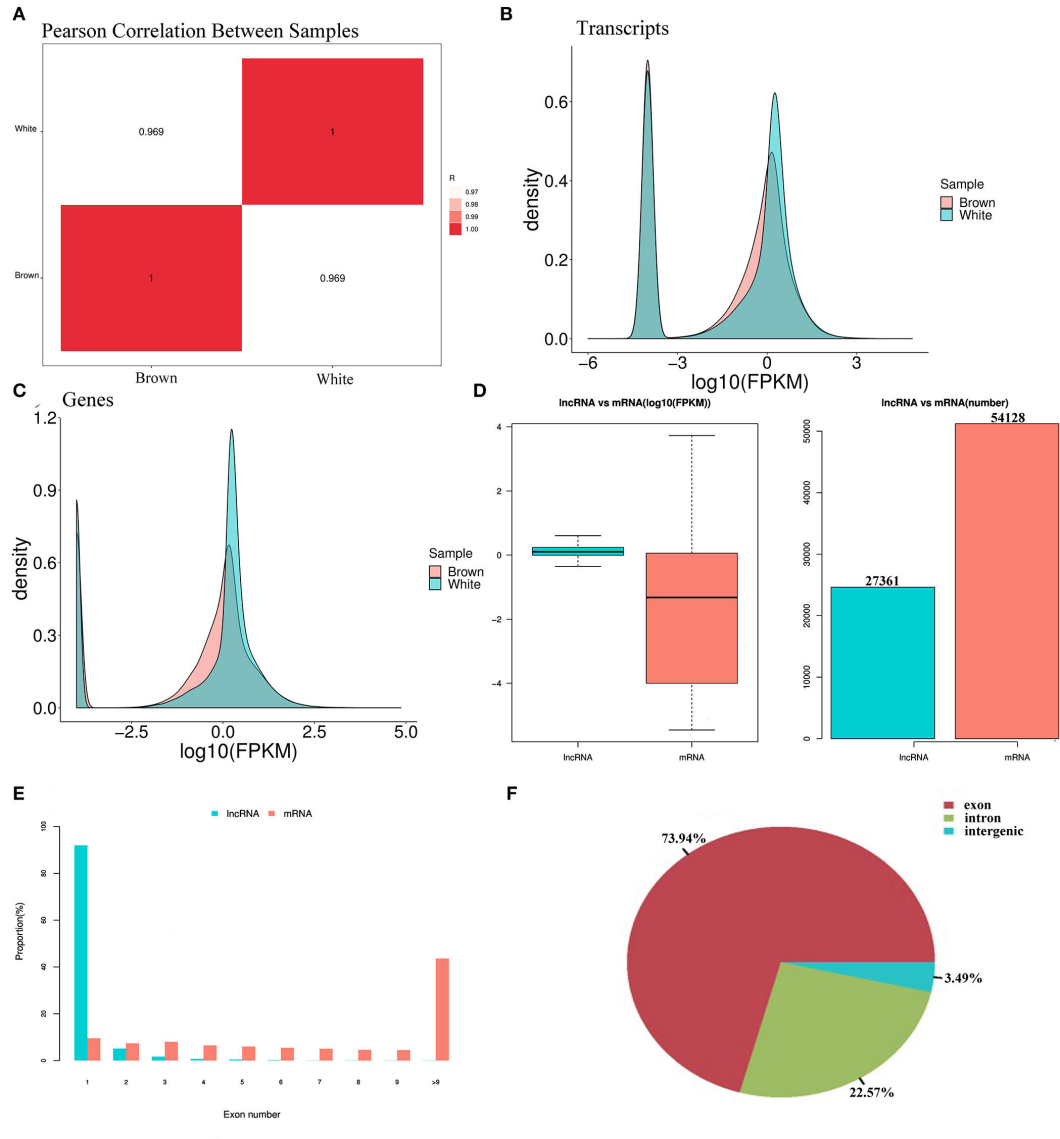
Expression patterns of lncRNA and mRNA in melanocytes isolated from Boer goat skins with white and brown hairs. **(A)** Pearson's correlation coefficient between different samples. **(B,C)** Expression value density of transcripts and gene expression trend. **(D)** Expression levels of lncRNAs and mRNAs in melanocytes. **(E)** Distribution of lncRNA and mRNA length in melanocytes. **(F)** Distribution of lncRNA located in melanocytes.

When comparing melanocytes isolated from Boer goat skins with white and brown hairs, Pearson's correlation coefficient was 0.969, indicating a marked correlation (Figure 3A). Quality control analysis found that the expression density map of each sample followed a normal distribution, and the expression trends of biological replicates tended to be consistent (Figures 3B,C). Transcriptional analysis showed that there were 27,361 lncRNAs and 54,128 mRNAs in the two cell groups, and in melanocytes, the expression levels of mRNAs were significantly higher than those of lncRNAs (Figure 3D). When compared with mRNAs, most lncRNAs only had one exon, which may be due to the process of lncRNA formation (Figure 3E). Statistics showed that most lncRNAs in melanocytes were derived from exons and introns, with a small portion being derived from intergene regions (Figure 3F).

### Divergent expression patterns of mRNA

**Figure 4:**
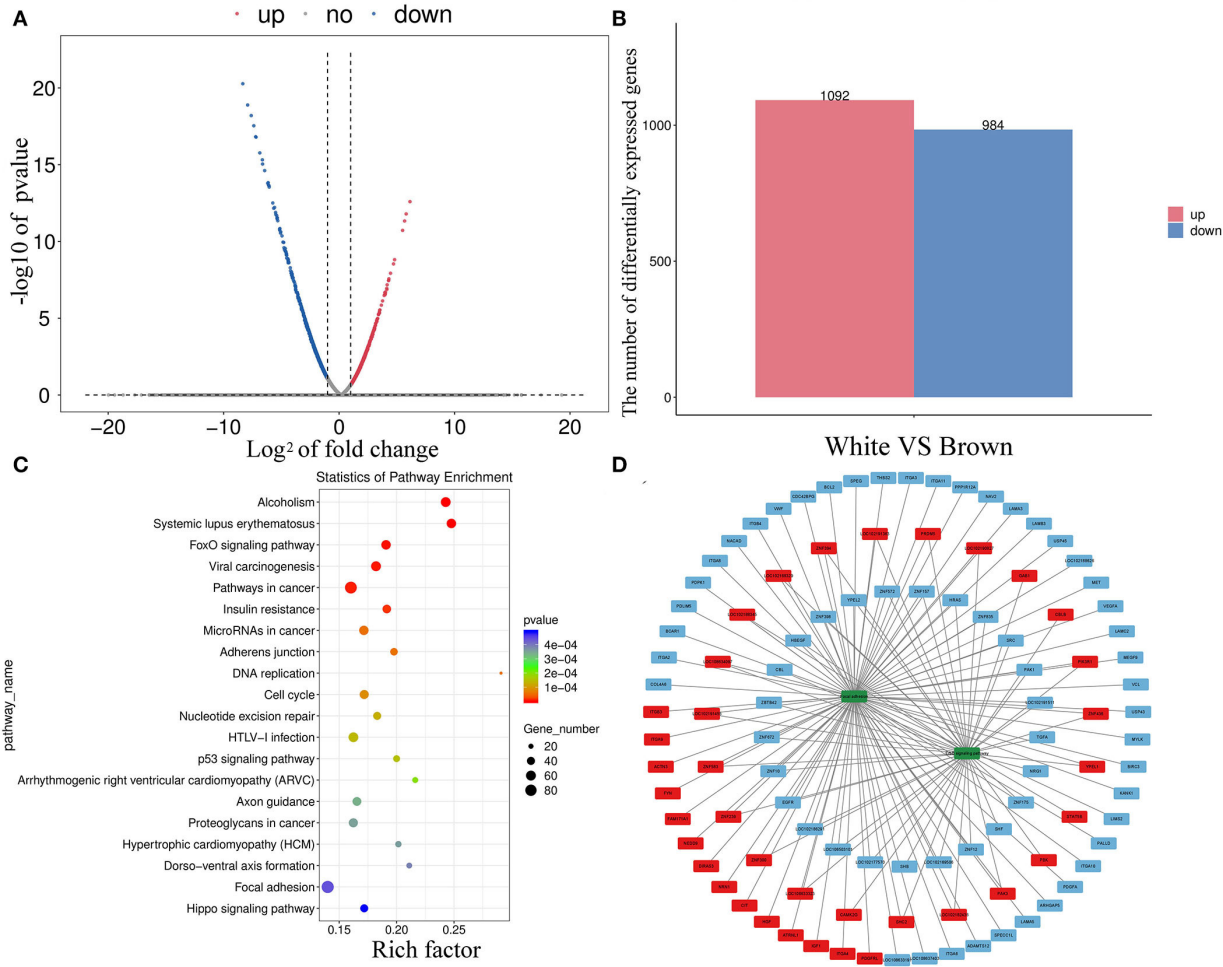
Divergent expression patterns of mRNAs in melanocytes isolated from Boer goat skins with white and brown hairs. **(A,B)** Divergent expression patterns of mRNAs in melanocytes. **(C,D)** DEG annotation results were obtained using the KEGG database; the red symbol marks positive regulatory genes, the blue marks negative regulatory genes, and the green marks key signaling pathway.

Transcriptome sequencing analysis found 2,076 DEGs between melanocytes isolated from goat skins with white and brown hairs (Figures 4A,B); the top 30 DEGs are shown in Supplementary Table S1. The KEGG database was used to annotate the 2,076 DEGs, and the top 20 KEGG annotation terms (KEGG term) were selected to generate a scatter diagram of the KEGG enrichment analysis (Figure 4C). The analysis found that the DEGs were significantly enriched in the ErbB signaling (32) and focal adhesion regulatory pathways (33), which are involved in tyrosinase activation and cell migration (Figure 4D).

### Divergent expression patterns of lncRNAs

**Figure 5:**
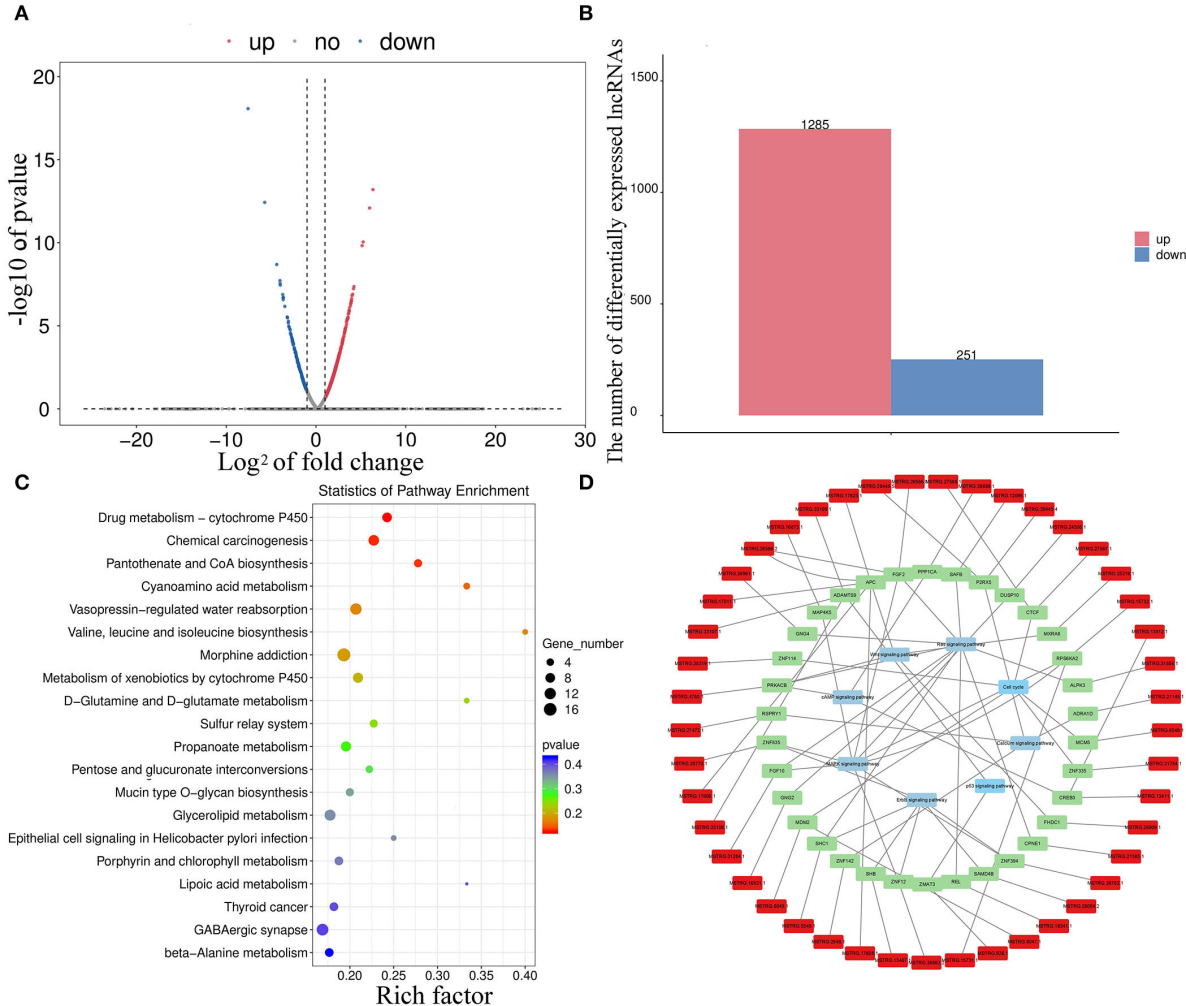
Divergent expression patterns of lncRNAs in melanocytes isolated from Boer goat skins with white and brown hairs. **(A,B)** Divergent expression patterns of lncRNAs in melanocytes. **(C,D)** The target gene of DEL annotation results was obtained using the KEGG database; red symbols mark DELs, green marks target genes of DELs, and blue marks key signaling pathways.

In total, 1,536 lncRNAs (251 were downregulated and 1,285 were upregulated) were differentially expressed in melanocytes isolated from goat skins with white and brown hairs (Figures 5A,B; Supplementary Table S2). Homology analysis showed that there were nine DELs with the same amino acid sequences as miRNA precursors and may correspond to miRNA precursors (Supplementary Table S3). lncRNA also has a potential *cis*-regulatory capacity for adjacent coding genes (34, 35). After the prediction of DEL *cis*-target genes (Supplementary Table S4), statistics from KEGG annotation found that the top potential target gene-enriched terms were involved in GABAergic synapse (Figure 5C), which may promote the expression and secretion of gonadotropin signaling molecules (36). Moreover, KEGG annotation revealed that the potential target genes of DELs were involved in the cAMP (37), MAPK (38), and ErbB signaling pathways (32), as well as other pigmentation-related pathways (Figure 5D).

### Identification of the core lncRNA–mRNA networks

**Figure 6:**
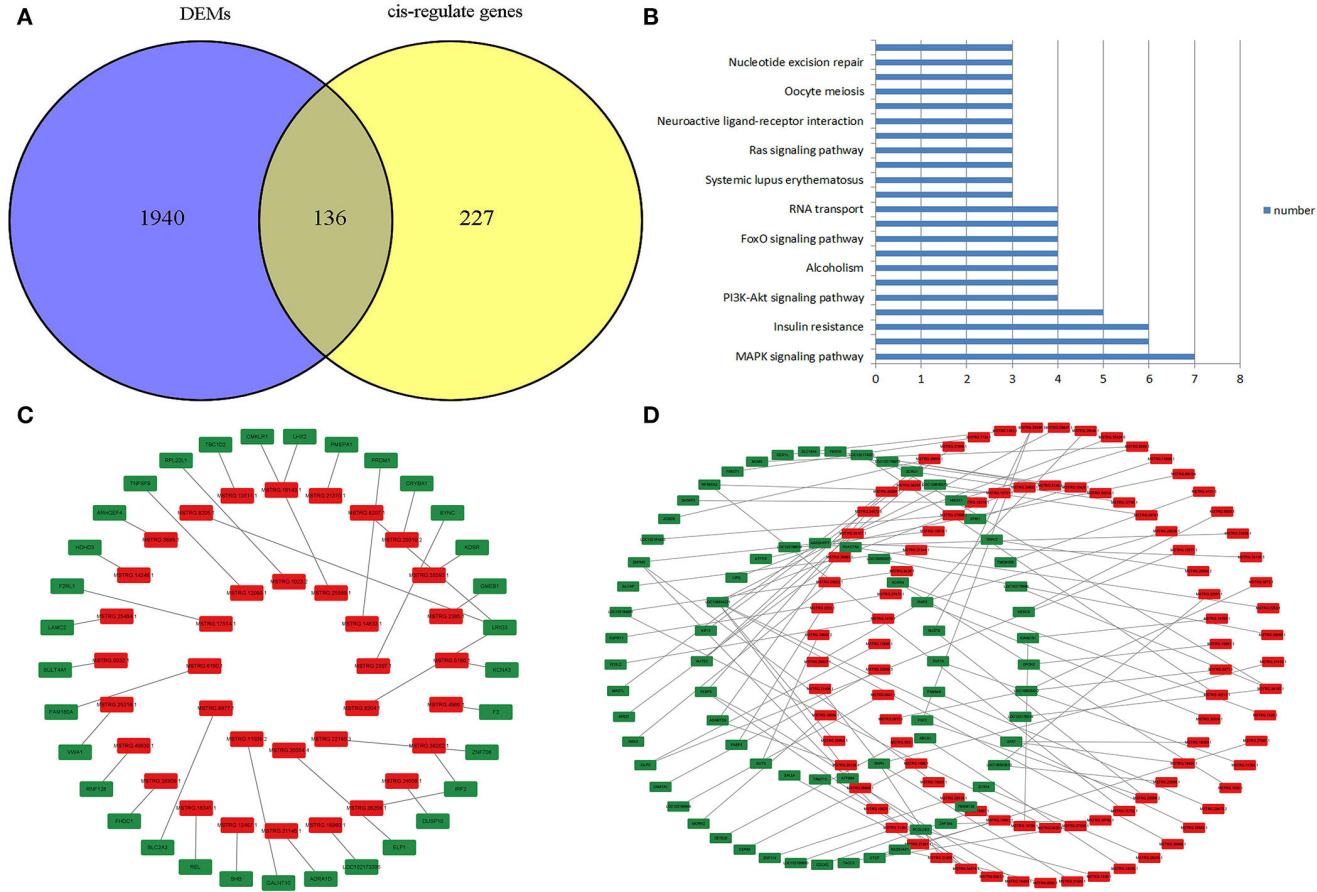
Identification of the core lncRNA–mRNA network. **(A)** Venn plot of *cis*-genes of DELs overlapped with DEMs. **(B)** Functional enrichment analysis of DELs that target DEMs. **(C)** Core negative regulatory DEL–DEM networks in melanocytes. **(D)** Core positive regulatory DEL–DEM networks in melanocytes; the red symbol marks the DELs, and green marks the DEMs.

Core networks were identified from common terms of *cis*-regulation of DELs and DEMs to analyze the molecular mechanisms underlying cell proliferation, migration, and melanogenesis of melanocytes. Statistics found that 136 *cis-*genes of DELs overlapped with DEMs (Figure 6A; Supplementary Table S5), and 139 DELs and *cis*-genes overlapped with DEMs with the same expression trends. KEGG annotation found that the top enriched terms of target DEMs were involved in the MAPK signaling pathway (Figure 6B), and thus, the core DEL–DEM networks were constructed (Figures 6C,D).

## Discussion

In this study, melanocytes from goat skins were successfully isolated and verified *in vitro*. The morphological analysis found that melanocytes isolated from goat skins with white hair were spindle-shaped with a short protrusion, while the number and the length of protrusions in melanocytes isolated from skins with brown hair were increased, which may be related to melanin transport.

Phenotypic testing revealed that melanocytes isolated from goat skins with white and brown hairs showed significant differences in proliferation, migration, and melanogenesis. To analyze the molecular mechanisms underlying phenotypic differences in the two groups, we determined the expression profiles of lncRNAs and mRNAs. According to the transcriptome data analysis, there were 2,076 DEMs in melanocytes isolated from goat skins with white and brown hairs. This suggests that these DEGs were possibly related to regulating melanocyte proliferation, migration, and melanogenesis. The ErbB signaling pathway (32) and the focal adhesion regulatory pathway (33) are key for regulating tyrosinase activation and cell migration, and the KEGG annotation results showed that there were multiple DEGs located in these two regulatory pathways. For example, ITGA6 is significantly overexpressed in hepatocellular carcinoma and mediates tumor progression (39). ESRRG may promote the proliferation and migration of Ishikawa cells (40). FGF2 stimulates the growth of trunk neural crest cells and promotes melanocytic commitment (41). Thus, DEGs can provide novel insights for analyzing the characteristics of melanocytes.

Long noncoding RNAs are associated with multiple physiological processes in animals [(21); 18]. In the present study, we found 27,361 novel potential lncRNAs in melanocytes. The expression of lncRNAs usually causes tissue- and stage-specific patterns in animals (30). Thus, the abundance profiles identified were assumed to be candidate melanocyte-specific lncRNAs. Currently, the functions of lncRNAs are investigated through their target genes, which may be regulated by *cis*-regulatory methods as previously described (34). In the present study, we predicted the *cis*-target genes of DELs and annotated the functions of potential target genes using the KEGG databases. The annotation results found that multiple predicted target genes were involved in MAPK signaling and the regulation of the actin cytoskeleton signaling pathway, which are closely related to cell proliferation, migration, and Melanogenesis. For example, FGF10 may upregulate cell proliferation in white adipose tissues (42), and APCs are widely involved in physiological processes such as cell proliferation, migration, and melanin production (43). These results suggest that the related DELs may participate in the physiological processes by regulating target genes. lncRNAs may also act as miRNA precursors to develop mature miRNAs and exert their function through targeted adsorption of downstream genes (44). The comparison of DEL sequences revealed that nine DELs had high homology with miRNA precursors, which may be their respective precursors. It has been reported that let-7e-5p and miR-574-5p are involved in the proliferation and migration of a variety of cells (45).

The *cis*-regulatory effects of lncRNAs on neighboring genes have been well-examined (30, 35). In this study, 136 *cis*-genes of DELs overlapped with DEMs, and 139 paired DELs and *cis*-genes had the same expression trends. KEGG annotation statistics found that the top enriched terms of the target DEMs were involved in the MAPK signaling pathway, which is the key pathway for regulating proliferation, migration, and melanogenesis, and thus, the DEL-DEM networks were constructed.

## Conclusion

In the study, melanocytes were successfully isolated from Boer goat skins *in vitro*. Phenotypic testing revealed that melanocytes isolated from goat skins with white and brown hair showed significant differences in cell proliferation, migration, and melanogenesis (^**^P < 0.01). Through prediction analysis, genome-wide stage-specific candidate lncRNAs were identified in goat melanocytes. DELs and DEGs in melanocytes isolated from goat skins were screened, and the functions of the source genes of the DELs and DEMs were annotated. The functions of DELs in *cis*-regulation and miRNA precursor functions were investigated. Based on our results, multiple lncRNA–mRNA networks may be involved in key signaling pathways in melanocyte proliferation, migration, and melanin production. This research provided novel bioinformatic insights into the roles of lncRNAs in mammalian pigmentation.

## Data availability statement

The datasets for this study can be found in the NCBI database and the BioProject accession number is PRJNA865095.

## Ethics statement

The animal study was reviewed and approved by the Ethics Committee of Anhui Agricultural University.

## Author contributions

JK-y and ZY-h: conceptualization, investigation, data analysis, and writing-original draft preparation. WR-j, IK, and ZY-W: methodology. All authors have read and approved the final manuscript.

## Funding

This study was funded by the Anhui Provincial Natural Science Foundation (grant number 2008085QC158), the University Natural Science Research Project of Anhui Province (grant number KJ2019A0165), and Anhui Provincial Natural Science Foundation (grant number 1908085QC144).

## Conflict of interest

The authors declare that the research was conducted in the absence of any commercial or financial relationships that could be construed as a potential conflict of interest.

## Publisher's note

All claims expressed in this article are solely those of the authors and do not necessarily represent those of their affiliated organizations, or those of the publisher, the editors and the reviewers. Any product that may be evaluated in this article, or claim that may be made by its manufacturer, is not guaranteed or endorsed by the publisher.
