# Supplementary material for: A genome-wide integrated analysis of lncRNA-mRNA in melanocytes from white and brown skin hair boer goats (Capra aegagrus hircus)
Source: Front Vet Sci. 2022 Nov 3;9:1009174. doi: 10.3389/fvets.2022.1009174 (PMC9669430; doi:10.3389/fvets.2022.1009174)
Supplement: Supplementary file 1 [file Data_Sheet_1.docx]

Supplementary Material

**Supplementary Table 1** Top 30 DEGs in melanocytes isolated from Boer goat white and brown skins.

| gene_name | Pval | log2(fc) | gene_name | Pval | log2(fc) |
| --- | --- | --- | --- | --- | --- |
| ITGA6 | 0.00 | -8.34 | ESRRG | 0.00 | 6.14 |
| CCDC3 | 0.00 | -7.92 | TP53INP2 | 0.00 | 5.81 |
| KRT5 | 0.00 | -7.61 | CLTA | 0.00 | 5.67 |
| GJB2 | 0.00 | -7.39 | SCG2 | 0.00 | 5.50 |
| LOC108633191 | 0.00 | -7.22 | ZBTB20 | 0.00 | 4.81 |
| DSG3 | 0.00 | -7.19 | LOC102171414 | 0.00 | 4.71 |
| ABLIM1 | 0.00 | -6.87 | TOR1AIP1 | 0.00 | 4.45 |
| ITGB4 | 0.00 | -6.64 | ACKR4 | 0.00 | 4.31 |
| TP63 | 0.00 | -6.63 | RRP36 | 0.00 | 4.30 |
| NRG1 | 0.00 | -6.45 | MRPS10 | 0.00 | 4.28 |
| DSP | 0.00 | -6.18 | SPIN1 | 0.00 | 4.16 |
| COL17A1 | 0.00 | -6.14 | ZNF436 | 0.00 | 4.14 |
| ABCG1 | 0.00 | -6.11 | SNX10 | 0.00 | 4.13 |
| KRT14 | 0.00 | -6.07 | MX1 | 0.00 | 4.02 |
| KRT8 | 0.00 | -6.05 | LOC102179149 | 0.00 | 4.01 |
| L1CAM | 0.00 | -5.74 | HOXB9 | 0.00 | 3.97 |
| NDUFA4L2 | 0.00 | -5.66 | LIPG | 0.00 | 3.96 |
| LAMA3 | 0.00 | -5.59 | ATF7IP | 0.00 | 3.90 |
| CELSR1 | 0.00 | -5.50 | SERBP1 | 0.00 | 3.76 |
| CXCL14 | 0.00 | -5.48 | OAF | 0.00 | 3.66 |
| LAMC2 | 0.00 | -5.40 | SP1 | 0.00 | 3.53 |
| TNS4 | 0.00 | -5.38 | WWTR1 | 0.00 | 3.52 |
| LAMB3 | 0.00 | -5.36 | LOC102179192 | 0.00 | 3.50 |
| SESN3 | 0.00 | -5.36 | PFDN1 | 0.00 | 3.48 |
| LOC102177275 | 0.00 | -5.35 | ADD2 | 0.00 | 3.44 |
| SFN | 0.00 | -5.31 | PEG10 | 0.00 | 3.42 |
| WIPF1 | 0.00 | -5.15 | PACSIN2 | 0.00 | 3.41 |
| LAMP3 | 0.00 | -5.14 | OSER1 | 0.00 | 3.34 |
| PMEPA1 | 0.00 | -5.13 | LOC102184572 | 0.00 | 3.30 |
| EPHA1 | 0.00 | -5.07 | KCNJ15 | 0.00 | 3.29 |

**Supplementary Table 2** The list of DELs in melanocytes isolated from Boer goat white and brown skins.

| Host gene name | LncRNA name | strand | start | end | log2(fc) | pval |
| --- | --- | --- | --- | --- | --- | --- |
| . | MSTRG.41560.3 | - | 775 | 3316 | -7.57 | 0.00 |
| . | MSTRG.30145.2 | + | 42906323 | 42933308 | -5.71 | 0.00 |
| RPL22L1 | MSTRG.1023.2 | + | 96122859 | 96125538 | -4.37 | 0.00 |
| . | MSTRG.30144.2 | - | 42876599 | 42891351 | -4.01 | 0.00 |
| . | MSTRG.27054.1 | - | 7938091 | 7940669 | -4.00 | 0.00 |
| . | MSTRG.12970.1 | . | 22538489 | 22541846 | -3.98 | 0.00 |
| NONO | MSTRG.41083.1 | - | 15718 | 18555 | -3.71 | 0.00 |
| . | MSTRG.42017.3 | + | 2211 | 11929 | -3.68 | 0.00 |
| SRP14 | MSTRG.17021.2 | + | 67372084 | 67375360 | -3.65 | 0.00 |
| DPT | MSTRG.24737.2 | - | 34820378 | 34856687 | -3.63 | 0.00 |
| LOC102168973 | MSTRG.6190.1 | + | 20870564 | 20871896 | -3.48 | 0.00 |
| SAP30L | MSTRG.11035.2 | - | 44641091 | 44650199 | -3.20 | 0.00 |
| ZNF706 | MSTRG.22186.3 | - | 19031763 | 19039342 | -3.18 | 0.00 |
| LOC102188814 | MSTRG.34320.4 | - | 21484811 | 21489921 | -3.09 | 0.00 |
| TAF1D | MSTRG.39092.12 | + | 592419 | 594267 | -3.07 | 0.00 |
| FAM78B | MSTRG.5799.1 | . | 117415544 | 117417336 | 3.85 | 0.00 |
| LOC102188534 | MSTRG.31627.21 | + | 1206744 | 1283333 | 3.86 | 0.00 |
| . | MSTRG.31284.1 | . | 30464978 | 30469341 | 3.90 | 0.00 |
| ANGPT1 | MSTRG.22312.1 | . | 25175601 | 25182215 | 3.91 | 0.00 |
| ANGPT1 | MSTRG.22306.1 | . | 25132077 | 25138423 | 3.92 | 0.00 |
| FAM78B | MSTRG.5796.1 | . | 117401753 | 117405023 | 3.95 | 0.00 |
| SESTD1 | MSTRG.3742.4 | - | 118606372 | 118649871 | 3.97 | 0.00 |
| . | MSTRG.39624.1 | + | 44032732 | 44097776 | 4.00 | 0.00 |
| ANGPT1 | MSTRG.22307.1 | . | 25140542 | 25145688 | 4.08 | 0.00 |
| TAX1BP3 | MSTRG.29445.4 | - | 24167062 | 24171943 | 4.18 | 0.00 |
| . | MSTRG.41871.1 | . | 3305 | 3609 | 4.23 | 0.00 |
| . | MSTRG.24477.1 | . | 19120906 | 19131183 | 5.13 | 0.00 |
| ATF7IP | MSTRG.8919.4 | - | 93982452 | 93986556 | 5.25 | 0.00 |
| . | MSTRG.24466.1 | . | 19048176 | 19057242 | 5.97 | 0.00 |
| . | MSTRG.24465.1 | . | 19033115 | 19047706 | 6.34 | 0.00 |

**Supplementary Table 3** The List of prediction results of miRNA precursor prediction

| miRNA | Forward(+)/  reverse(-) | lncRNA | start | end | pre-miRNA-seq |
| --- | --- | --- | --- | --- | --- |
| miR-1285-p5-  1ss6TC | + | MSTRG.16889.3 | 209 | 313 | ACUCCCGUGCUGAUCAGUAGUGGGA  UCGCGCCUGUGAAUAGCCACUGUAC  UCCAGCCUGGGCAACGGAGCAGGUC  AAAACUCCCGUGCUGAUCAGUAGUG  GGAUC |
| miR-574-5p | - | MSTRG.1811.1 | 4358 | 4264 | UAACUCAUACAUUAUAUAUAUUCGU  AAGAACAAAAUAUUUAUAUACUUUA  CAUAUAUGUCCCAAAGUGUUCAUGA  GUGUGUGUGUGUGUGUGUGU |
| let-7e-5p | + | MSTRG.23763.1 | 1649 | 1713 | UGAGGUAGUAGGUUGUAUAGUUUAG  AAUUACAUCAAGGGAGAUAACUGUA  CAGCCUCCUAGCUUU |
| PC-5p-30128_5 | + | MSTRG.26857.1 | 3577 | 3635 | UGCCUAGCUCUCACCCCCCAGUCAGU  CUGAUUUCAUUGCUGUUGGGUGUGA  ACUAGGAA |
| miR-2284y-1-p5 | - | MSTRG.29706.1 | 116 | 61 | AAAAGUUAGUUCGGGUUUUCCUGUA  AGAUGUUGUGGAAAAACCUGAAUGA  ACUUUC |
| miR-574-5p | - | MSTRG.32102.1 | 137 | 70 | UGUGUGUGUGUGUGUGUGUGUGUAU  AGUGUAUCUCACAUGCUAAGACAGG  UGCAUUCCACAUACACCA |
| miR-2284y | - | MSTRG.33106.1 | 12322 | 12268 | AAAAGUUCAUUCGGGUUUUCCAGCC  CAUCUUACAGAAAAACCCAAACAAA  UCUUU |
| PC-5p-26755_6 | + | MSTRG.8106.1 | 5402 | 5466 | GAAUUAAUGGCUGGCUGGGAGGCAG  AGCCAAGGGCCACUGGUUCCUCCCA  GCUGGUCAUUAAUCC |
| miR-2284y-1-p5 | - | MSTRG.9420.1 | 384 | 324 | AAAAGUUCAUUCUGGUUUUUCCAUU  CCAUAAAAUCUUAUGGAAAAACUGG  AACAAACUUUU |

**Supplementary Table 4** The List of prediction results of cis-target genes with DELs in melanocytes isolated from Boer goat white and brown skins.

| mRNA | mRNA | lncRNA |  |  |  |  |
| --- | --- | --- | --- | --- | --- | --- |
| transcript_name | gene_name | transcript_name | start | end | Host gene_name | cis location |
| XM_005675607.3 | ACKR4 | MSTRG.1532.1 | 136607126 | 136613987 | ACAD11 | 10K |
| XM_005695759.3 | ADAMTS9 | MSTRG.33109.1 | 36793181 | 36796522 | ADAMTS9 | 100K |
| XM_005695759.3 | ADAMTS9 | MSTRG.33107.1 | 36769408 | 36771945 | ADAMTS9 | 100K |
| XM_005695759.3 | ADAMTS9 | MSTRG.33106.1 | 36756464 | 36768956 | ADAMTS9 | 10K |
| XM_005688233.3 | ADRA1D | MSTRG.21145.1 | 50297181 | 50299650 | . | 100K |
| XM_018042688.1 | AHNAK | MSTRG.39547.5 | 41211874 | 41217228 | AHNAK | 10K |
| XM_018042918.1 | ALDH3B1 | MSTRG.39769.1 | 46588917 | 46590863 | ALDH3B1 | 10K |
| XM_005685405.2 | APEX1 | MSTRG.17124.1 | 76343923 | 76360074 | LOC102176691 | 100K |
| XM_018048451.1 | ATF7IP | MSTRG.8919.4 | 93982452 | 93986556 | ATF7IP | 10K |
| XM_018058513.1 | ATP6V1H | MSTRG.22647.1 | 60130175 | 60132810 | . | 100K |
| XM_018054182.1 | ATP8B4 | MSTRG.16490.1 | 42709755 | 42712491 | . | 100K |
| XM_018040073.1 | AUTS2 | MSTRG.36289.1 | 29699144 | 29702984 | AUTS2 | 100K |
| XM_018048707.1 | BCL2L13 | MSTRG.9142.9 | 108006818 | 108043889 | BCL2L13 | 1K |
| XM_018048714.1 | BID | MSTRG.9142.9 | 108006818 | 108043889 | BCL2L13 | 100K |
| XM_018038994.1 | C23H6orf62 | MSTRG.34100.5 | 16909207 | 16913208 | C23H6orf62 | 10K |
| XM_018039646.1 | CCBE1 | MSTRG.35565.2 | 58592217 | 58597093 | CCBE1 | 100K |
| XM_018046057.1 | CERS2 | MSTRG.5447.1 | 100429665 | 100437260 | CDC42SE1 | 100K |
| XM_005691599.3 | CMKLR1 | MSTRG.25989.1 | 6511363 | 6514274 | . | 10K |
| XM_018064425.1 | CTNS | MSTRG.29445.4 | 24167062 | 24171943 | TAX1BP3 | 100K |
| XR_001917494.1 | CTSC | MSTRG.39112.1 | 6800469 | 6803721 | . | 10K |
| XM_018053039.1 | DSE | MSTRG.14666.1 | 21555752 | 21557903 | DSE | 100K |
| XM_013966362.2 | ECHDC1 | MSTRG.14555.1 | 11265073 | 11266009 | ECHDC1 | 100K |
| XM_018056807.1 | ELF1 | MSTRG.20354.4 | 75256953 | 75261114 | WBP4 | 10K |
| XM_018042693.1 | EML3 | MSTRG.39547.5 | 41211874 | 41217228 | AHNAK | 100K |
| XM_018053694.1 | F2RL1 | MSTRG.17514.1 | 93176210 | 93179537 | . | 10K |
| XM_005679506.3 | FAM180A | MSTRG.6190.1 | 20870564 | 20871896 | LOC102168973 | 100K |
| XM_005696773.3 | FAM65B | MSTRG.34100.5 | 16909207 | 16913208 | C23H6orf62 | 100K |
| XM_005677089.2 | FAM78B | MSTRG.5796.1 | 117401753 | 117405023 | FAM78B | 100K |
| XM_005677089.2 | FAM78B | MSTRG.5799.1 | 117415544 | 117417336 | FAM78B | 100K |
| XM_005677089.2 | FAM78B | MSTRG.5800.1 | 117418057 | 117428201 | FAM78B | 100K |
| XM_005677089.2 | FAM78B | MSTRG.5797.1 | 117405705 | 117409048 | FAM78B | 100K |
| XM_005677089.2 | FAM78B | MSTRG.5803.1 | 117445728 | 117450926 | FAM78B | 100K |
| XM_005677089.2 | FAM78B | MSTRG.5802.1 | 117436474 | 117444990 | FAM78B | 100K |
| XM_005677089.2 | FAM78B | MSTRG.5804.1 | 117451179 | 117452296 | FAM78B | 100K |
| XM_018055205.1 | FBXO11 | MSTRG.18260.2 | 29714934 | 29718736 | FBXO11 | 1K |
| XM_005694730.3 | FGF10 | MSTRG.31284.1 | 30464978 | 30469341 | . | 10K |
| XM_018061205.1 | FGF2 | MSTRG.26586.3 | 36267841 | 36278256 | FGF2 | 100K |
| XM_018061487.1 | FNIP2 | MSTRG.26537.1 | 30851257 | 30852761 | FNIP2 | 100K |
| XM_018043569.1 | FSTL1 | MSTRG.542.1 | 64829638 | 64880899 | FSTL1 | 1K |
| XM_018050128.1 | GALNT10 | MSTRG.11035.2 | 44641091 | 44650199 | SAP30L | 100K |
| XM_005699778.3 | GANAB | MSTRG.39547.5 | 41211874 | 41217228 | AHNAK | 100K |
| XM_005685401.3 | LOC102176691 | MSTRG.17124.1 | 76343923 | 76360074 | LOC102176691 | 100K |
| XR_001919884.1 | LOC102188534 | MSTRG.31639.1 | 1165107 | 1168090 | LOC102188534 | 10K |
| XM_005696612.3 | LOC102188814 | MSTRG.34320.4 | 21484811 | 21489921 | LOC102188814 | 1K |
| XR_001917466.1 | LOC108634221 | MSTRG.39855.1 | 49975673 | 49977289 | KCNQ1 | 10K |
| XM_018047999.1 | LRIG3 | MSTRG.8204.1 | 53868593 | 53869102 | LRIG3 | 100K |
| XM_018057739.1 | MAP1LC3A | MSTRG.21534.1 | 63488792 | 63492052 | . | 100K |
| XM_018047968.1 | MDM2 | MSTRG.8049.1 | 44340670 | 44349825 | . | 100K |
| XM_018060525.1 | MRPL20 | MSTRG.25216.1 | 49601623 | 49603856 | . | 100K |
| XM_018060531.1 | MXRA8 | MSTRG.25216.1 | 49601623 | 49603856 | . | 100K |
| XM_005681127.3 | NDUFA6 | MSTRG.9298.1 | 112070489 | 112072869 | LOC102169002 | 100K |
| XM_013976413.2 | NONO | MSTRG.41083.1 | 15718 | 18555 | NONO | 1K |
| XM_018061508.1 | NUDT6 | MSTRG.26586.3 | 36267841 | 36278256 | FGF2 | 100K |
| XM_018055754.1 | OLFML2A | MSTRG.19208.1 | 95221346 | 95224548 | NR6A1 | 100K |
| XM_018064429.1 | P2RX5 | MSTRG.29445.4 | 24167062 | 24171943 | TAX1BP3 | 10K |
| XM_005692399.3 | PLEKHG2 | MSTRG.28004.2 | 49969273 | 49972415 | MED29 | 100K |
| XM_005685402.3 | PNP | MSTRG.17124.1 | 76343923 | 76360074 | LOC102176691 | 10K |
| XM_018053117.1 | PRDM1 | MSTRG.14833.1 | 31125806 | 31127592 | PRDM1 | 100K |
| XM_018039016.1 | RAB23 | MSTRG.34772.1 | 45687472 | 45689072 | RAB23 | 100K |
| XM_018048513.1 | RIMKLB | MSTRG.8981.1 | 100184458 | 100184727 | . | 100K |
| XM_018052944.1 | RNF146 | MSTRG.14555.1 | 11265073 | 11266009 | ECHDC1 | 100K |
| XM_005675323.2 | RPL22L1 | MSTRG.1023.2 | 96122859 | 96125538 | RPL22L1 | 10K |
| XM_018062387.1 | SAMD4B | MSTRG.28004.2 | 49969273 | 49972415 | MED29 | 100K |
| XM_018046048.1 | SETDB1 | MSTRG.5447.1 | 100429665 | 100437260 | CDC42SE1 | 100K |
| XM_018053243.1 | SGO1 | MSTRG.1890.2 | 157179348 | 157185519 | KAT2B | 100K |
| XM_018041255.1 | SH3PXD2A | MSTRG.37415.1 | 27527166 | 27528267 | SH3PXD2A | 100K |
| XM_018041252.1 | SH3PXD2A | MSTRG.37393.4 | 27347086 | 27447777 | SH3PXD2A | 10K |
| XM_018041255.1 | SH3PXD2A | MSTRG.37413.1 | 27521275 | 27522879 | SH3PXD2A | 100K |
| XM_005680306.3 | SHMT2 | MSTRG.8237.2 | 55533680 | 55535985 | NDUFA4L2 | 10K |
| NM_001285741.1 | SLC2A3 | MSTRG.8977.1 | 99851317 | 99851866 | . | 100K |
| XM_005685480.3 | SRP14 | MSTRG.17021.2 | 67372084 | 67375360 | SRP14 | 1K |
| XR_001917230.1 | SRRM2 | MSTRG.35762.2 | 2174775 | 2176905 | SRRM2 | 1K |
| XM_005686514.3 | SRSF7 | MSTRG.18051.2 | 20926868 | 20930665 | SRSF7 | 1K |
| XM_018057857.1 | SYNC | MSTRG.2397.1 | 14740547 | 14740993 | SYNC | 10K |
| XM_018065313.1 | TLK2 | MSTRG.30268.1 | 46554290 | 46556457 | TLK2 | 100K |
| XM_018050941.1 | TNFSF9 | MSTRG.12090.1 | 92533600 | 92547742 | . | 10K |
| XM_013962597.2 | TRMT13 | MSTRG.5041.3 | 77325917 | 77333649 | SASS6 | 100K |
| XM_018043450.1 | UNC93B1 | MSTRG.39769.1 | 46588917 | 46590863 | ALDH3B1 | 100K |
| XM_018060522.1 | VWA1 | MSTRG.25216.1 | 49601623 | 49603856 | . | 10K |
| XM_005676696.3 | ZBTB8OS | MSTRG.2397.1 | 14740547 | 14740993 | SYNC | 100K |
| XM_018058317.1 | ZNF706 | MSTRG.22186.3 | 19031763 | 19039342 | ZNF706 | 10K |

**Supplementary Table 5** The List of cis-target genes of DELs overlapped with DEMs in melanocytes isolated from Boer goat white and brown skins.

| Target gene/DEMs | log2(fc) | DELs | log2(fc) |
| --- | --- | --- | --- |
| AASDHPPT | 1.01 | MSTRG.24169.3 | 2.059385454 |
| ABCA1 | 1.17 | MSTRG.14039.1 | 1.974808657 |
| ACBD5 | 1.17 | MSTRG.20636.1 | 1.455030469 |
| ACKR4 | 4.31 | MSTRG.1532.1 | 2.255654617 |
| ACKR4 | 4.31 | MSTRG.1526.1 | 1.539039854 |
| ADAMTS9 | 1.838426 | MSTRG.33109.1 | 2.46838324 |
| ADAMTS9 | 1.838426 | MSTRG.33107.1 | 2.412197574 |
| ADRA1D | -1.84612 | MSTRG.21145.1 | -1.793625265 |
| AP5Z1 | 1.15 | MSTRG.36883.1 | 1.51 |
| ARHGEF4 | -4.41 | MSTRG.3889.1 | -1.34104212 |
| ATF7IP | 3.90 | MSTRG.8919.4 | 5.25 |
| ATP8B4 | 2.64 | MSTRG.16490.1 | 3.41 |
| AUTS2 | 2.25 | MSTRG.36289.1 | 2.16 |
| AUTS2 | 2.25 | MSTRG.36283.1 | 1.91 |
| BLCAP | 2.36 | MSTRG.21644.1 | 1.07 |
| CACFD1 | 1.19 | MSTRG.19517.2 | -1.71 |
| CAMTA1 | 1.42808 | MSTRG.25090.3 | 1.217785509 |
| CDCA2 | 1.63 | MSTRG.13657.1 | 1.06 |
| CEP63 | 1.25 | MSTRG.1488.1 | 1.145317535 |
| CILP2 | 1.10 | MSTRG.12577.1 | 1.339738839 |
| CILP2 | 1.10 | MSTRG.12580.1 | 1.047293967 |
| CMKLR1 | -2.04 | MSTRG.25989.1 | -2.870133584 |
| CRYBA1 | -2.18 | MSTRG.29310.2 | -1.59 |
| CTCF | 1.870721 | MSTRG.27567.1 | 1.470591156 |
| CTCF | 1.870721 | MSTRG.27566.1 | 1.374462192 |
| CXHXorf23 | -1.38 | MSTRG.40341.1 | 1.10 |
| CXHXorf23 | -1.38 | MSTRG.40342.1 | 1.08 |
| DUSP10 | -1.46896 | MSTRG.24508.1 | -1.463823932 |
| DZIP1L | 1.471372 | MSTRG.1452.1 | 1.491994111 |
| ELF1 | -1.11363 | MSTRG.20354.4 | -2.153599166 |
| F2RL1 | -2.33 | MSTRG.17514.1 | -2.779255133 |
| F3 | -2.54 | MSTRG.4986.1 | -1.688369117 |
| FABP3 | 1.636318 | MSTRG.2333.1 | 1.789632172 |
| FAM180A | -3.50 | MSTRG.6190.1 | -3.48 |
| FAM64A | 1.76 | MSTRG.29473.2 | 2.02 |
| FAM65B | 1.800661 | MSTRG.34100.5 | -2.915961612 |
| FBXO5 | 1.42 | MSTRG.15425.1 | 1.113382657 |
| FGF10 | 3.04 | MSTRG.31284.1 | 3.896832305 |
| FGF2 | 1.964433 | MSTRG.26586.3 | 2.359082913 |
| FGF2 | 1.964433 | MSTRG.26586.2 | 1.338362295 |
| FHDC1 | -3.78 | MSTRG.26909.1 | -1.066781698 |
| FKBP5 | 1.22 | MSTRG.34674.1 | 2.02361238 |
| FKBP5 | 1.22 | MSTRG.34674.1 | 2.02361238 |
| FKBP5 | 1.22 | MSTRG.34673.5 | 1.667631782 |
| FNIP2 | 1.20 | MSTRG.26545.1 | 1.99 |
| FNIP2 | 1.20 | MSTRG.26537.1 | 2.16 |
| FNIP2 | 1.20 | MSTRG.26535.1 | 1.38 |
| FNIP2 | 1.20 | MSTRG.26536.1 | 1.38 |
| FOXF1 | -1.30806 | MSTRG.27186.1 | 1.145012319 |
| FOXL2 | 2.33 | MSTRG.1418.1 | 1.415475834 |
| FUBP1 | 1.20 | MSTRG.4805.1 | -1.05 |
| FUBP1 | 1.20 | MSTRG.4801.1 | -1.56 |
| GALNT10 | -1.40386 | MSTRG.11035.2 | -3.201349601 |
| GMEB1 | -1.95 | MSTRG.2285.1 | -1.462792291 |
| GNG4 | 1.40 | MSTRG.38961.1 | -1.246375622 |
| GOPC | -2.28 | MSTRG.14659.1 | 1.176329403 |
| GPR135 | 1.06 | MSTRG.16305.1 | -1.24 |
| HDHD3 | -1.11681 | MSTRG.14246.1 | -1.091014959 |
| HELQ | -1.16614 | MSTRG.10406.2 | 1.754400002 |
| HERC6 | 1.886384 | MSTRG.9809.1 | 1.48345876 |
| HID1 | -2.45 | MSTRG.30668.1 | 1.31 |
| HMOX1 | 1.621735 | MSTRG.8549.1 | 1.543762454 |
| IRF2 | -2.77 | MSTRG.38262.1 | -1.168289241 |
| IRF2 | -2.77 | MSTRG.38256.1 | -1.017633479 |
| ISYNA1 | -3.05 | MSTRG.12505.1 | 1.031630419 |
| KCNA3 | -1.04 | MSTRG.5160.1 | -1.284760689 |
| KDSR | -1.58984 | MSTRG.35593.1 | -1.108591584 |
| KIAA0101 | 2.54 | MSTRG.16789.1 | 1.06 |
| KIAA0101 | 2.54 | MSTRG.16788.1 | 1.34 |
| KIF11 | 1.680086 | MSTRG.37689.1 | 1.997253022 |
| LAMC2 | -5.40 | MSTRG.25484.1 | -1.530874172 |
| LHX2 | -2.37 | MSTRG.19143.1 | -1.098257496 |
| LIPG | 3.96 | MSTRG.35428.6 | 1.885484704 |
| LOC102173396 | -1.45087 | MSTRG.18960.1 | -1.304796702 |
| LOC102174355 | 1.15 | MSTRG.36213.1 | 1.091098325 |
| LOC102174355 | 1.15 | MSTRG.36214.1 | 1.069112858 |
| LOC102178318 | 2.093902 | MSTRG.34160.1 | 1.641933951 |
| LOC102178584 | 2.77 | MSTRG.34160.1 | 1.641933951 |
| LOC102178870 | 2.88 | MSTRG.34160.1 | 1.641933951 |
| LOC102181372 | -1.57 | MSTRG.9073.5 | 1.62 |
| LOC102181420 | 1.03 | MSTRG.27988.1 | 1.26 |
| LOC102184901 | 1.829291 | MSTRG.9420.1 | 1.271731527 |
| LOC102184901 | 1.829291 | MSTRG.9428.1 | 1.117014158 |
| LOC102188534 | 1.18 | MSTRG.31639.1 | 2.26 |
| LOC102189649 | 1.26 | MSTRG.6441.1 | 1.27 |
| LOC102190960 | 1.964185 | MSTRG.11291.1 | 1.595968557 |
| LOC106502810 | 1.05 | MSTRG.22990.1 | 1.06 |
| LOC106502810 | 1.05 | MSTRG.22991.1 | 1.11 |
| LOC106503675 | 1.19 | MSTRG.38060.1 | 1.88 |
| LOC108633863 | -1.80466 | MSTRG.36755.1 | 1.102844721 |
| LOC108634221 | 1.32 | MSTRG.39847.1 | 2.07 |
| LOC108634221 | 1.32 | MSTRG.39842.1 | 1.85 |
| LOC108634221 | 1.32 | MSTRG.39846.1 | 1.63 |
| LOC108634221 | 1.32 | MSTRG.39855.1 | 2.20 |
| LOC108634221 | 1.32 | MSTRG.39845.1 | 1.46 |
| LOC108634221 | 1.32 | MSTRG.39853.1 | 1.40 |
| LOC108635575 | 1.45 | MSTRG.4919.1 | 1.26 |
| LOC108635612 | 1.37 | MSTRG.5872.1 | 1.30 |
| LOC108635612 | 1.37 | MSTRG.5871.1 | 1.25 |
| LOC108635612 | 1.37 | MSTRG.5873.1 | 1.43 |
| LPCAT2 | -1.88 | MSTRG.27419.1 | 1.780441963 |
| LPCAT2 | -1.88 | MSTRG.27418.1 | 1.46695949 |
| LRIG3 | -2.07 | MSTRG.8204.1 | -2.08248073 |
| LRIG3 | -2.07 | MSTRG.8205.1 | -1.547745835 |
| LRIG3 | -2.07 | MSTRG.8207.1 | -1.23640056 |
| MASTL | 1.470527 | MSTRG.20636.1 | 1.455030469 |
| MCM5 | 1.16 | MSTRG.8549.1 | 1.543762454 |
| MORN2 | 1.28 | MSTRG.18064.1 | 1.69 |
| MPP5 | -2.42 | MSTRG.16187.2 | 1.519051727 |
| NEK2 | 1.41 | MSTRG.25622.1 | 1.576105619 |
| NT5C3A | 1.17 | MSTRG.6694.1 | -1.08229001 |
| NUDT6 | 2.87 | MSTRG.26586.3 | 2.36 |
| NUDT6 | 2.87 | MSTRG.26586.2 | 1.34 |
| NUP210 | -2.26 | MSTRG.33642.1 | 1.45 |
| OCIAD2 | -1.73 | MSTRG.10035.5 | 1.07 |
| OLFML2A | -1.97 | MSTRG.19208.1 | 2.10867733 |
| OLFML2A | -1.97 | MSTRG.19218.1 | 1.291428111 |
| OLFML2A | -1.97 | MSTRG.19206.1 | 1.658284306 |
| OLFML2A | -1.97 | MSTRG.19215.1 | 1.003305539 |
| PAPLN | -2.71 | MSTRG.16062.1 | 1.329876751 |
| PCOLCE2 | 1.420732 | MSTRG.1330.1 | 1.725134448 |
| PHACTR4 | 2.65 | MSTRG.2263.1 | 1.98 |
| PMEPA1 | -5.13 | MSTRG.21370.1 | -1.42 |
| PPP1R3G | -1.5477 | MSTRG.34084.1 | 1.894809379 |
| PRDM1 | -2.29 | MSTRG.14833.1 | -1.934134142 |
| RAD51AP1 | 2.46 | MSTRG.9052.1 | 1.47 |
| REL | -1.47 | MSTRG.18341.1 | -1.50 |
| RNF128 | -3.57 | MSTRG.40830.1 | -1.32 |
| RPL22L1 | -1.05 | MSTRG.1023.2 | -4.373790456 |
| RPS6KA2 | 1.34 | MSTRG.15732.1 | 1.148380038 |
| RPS6KA2 | 1.34 | MSTRG.15731.1 | 1.034445109 |
| RSPRY1 | 1.03 | MSTRG.27472.1 | 1.353730783 |
| SALL4 | 1.34 | MSTRG.21901.1 | 1.80567928 |
| SCRG1 | 1.21 | MSTRG.12790.1 | 1.28 |
| SGO1 | 1.34 | MSTRG.1890.2 | -2.624165916 |
| SH3RF3 | 1.08 | MSTRG.18379.1 | 1.21462946 |
| SHB | -1.92 | MSTRG.13467.1 | -1.203746363 |
| SLC16A4 | 1.06 | MSTRG.5149.1 | 1.343593976 |
| SLC2A3 | -2.38 | MSTRG.8977.1 | -2.272268672 |
| SLIT3 | 1.724896 | MSTRG.30805.2 | 2.001184918 |
| SNPH | 2.89 | MSTRG.21403.1 | 1.935357311 |
| SNPH | 2.89 | MSTRG.21410.1 | 1.728573972 |
| SNPH | 2.89 | MSTRG.21406.1 | 1.933105941 |
| SNPH | 2.89 | MSTRG.21409.1 | 1.525845437 |
| SPIN1 | 4.16 | MSTRG.13959.1 | 1.44 |
| SPON2 | 1.13 | MSTRG.10661.3 | 1.28 |
| SPRED2 | -1.05 | MSTRG.18687.1 | 1.17 |
| SPRED2 | -1.05 | MSTRG.18685.1 | 1.07 |
| SULT4A1 | -1.27 | MSTRG.9332.1 | -1.21 |
| SURF2 | 1.10 | MSTRG.19517.2 | -1.71 |
| SYNC | -2.66 | MSTRG.2394.1 | 1.852771063 |
| SYNC | -2.66 | MSTRG.2397.1 | -3.032583003 |
| TACC3 | 1.16 | MSTRG.10601.1 | 1.225146385 |
| TBC1D2 | -1.3394 | MSTRG.13511.1 | -1.231273979 |
| TEF | 2.188909 | MSTRG.9271.2 | -1.297706228 |
| TMEM129 | 1.84 | MSTRG.10601.1 | 1.23 |
| TMEM35B | 2.30 | MSTRG.4101.3 | 1.75 |
| TNFSF9 | -3.75 | MSTRG.12090.1 | -1.900696461 |
| TRIM68 | -1.57234 | MSTRG.23470.1 | 1.494442108 |
| TRMT13 | 2.08 | MSTRG.5041.3 | 2.24 |
| TRPV2 | 1.623925 | MSTRG.29684.2 | 1.17532459 |
| TWIST1 | 1.29 | MSTRG.7124.1 | 1.757234318 |
| UXS1 | 2.181078 | MSTRG.18404.1 | 1.275694774 |
| UXS1 | 2.181078 | MSTRG.18393.1 | 1.150713026 |
| VWA1 | -3.32 | MSTRG.25216.1 | -1.79740356 |
| ZBTB20 | 4.81 | MSTRG.500.1 | 1.081787024 |
| ZC3H4 | 2.95 | MSTRG.28263.1 | 1.43 |
| ZEB1 | -1.3712 | MSTRG.20851.1 | 1.460308291 |
| ZEB1 | -1.3712 | MSTRG.20853.1 | 1.129921521 |
| ZMYM5 | 1.04 | MSTRG.19923.1 | 1.52 |
| ZMYM5 | 1.04 | MSTRG.19920.1 | 1.35 |
| ZMYM5 | 1.04 | MSTRG.19919.1 | 1.29 |
| ZMYM5 | 1.04 | MSTRG.19924.1 | 1.00 |
| ZNF114 | 1.34 | MSTRG.28319.1 | 1.42725608 |
| ZNF12 | -1.54343 | MSTRG.36863.2 | 1.919607489 |
| ZNF142 | 1.10 | MSTRG.2648.1 | 1.021481178 |
| ZNF394 | 1.28 | MSTRG.36782.1 | 1.085659992 |
| ZNF706 | -1.86 | MSTRG.22186.3 | -3.18 |
| ZNF835 | -2.49 | MSTRG.28770.1 | 1.984725557 |
